# Modulatory Effects of *Eschscholzia californica* Alkaloids on Recombinant GABA_A_ Receptors

**DOI:** 10.1155/2015/617620

**Published:** 2015-10-05

**Authors:** Milan Fedurco, Jana Gregorová, Kristýna Šebrlová, Jana Kantorová, Ondřej Peš, Roland Baur, Erwin Sigel, Eva Táborská

**Affiliations:** ^1^Michelin Recherche et Technique S.A., Route André-Piller 30, 1762 Givisiez, Switzerland; ^2^Department of Biochemistry, Faculty of Medicine, Masaryk University, 62500 Brno, Czech Republic; ^3^Institute of Biochemistry and Molecular Medicine, University of Bern, Bühlstrasse 28, 3012 Bern, Switzerland

## Abstract

The California poppy (*Eschscholzia californica* Cham.) contains a variety of natural compounds including several alkaloids found exclusively in this plant. Because of the sedative, anxiolytic, and analgesic effects, this herb is currently sold in pharmacies in many countries. However, our understanding of these biological effects at the molecular level is still lacking. Alkaloids detected in *E. californica* could be hypothesized to act at GABA_A_ receptors, which are widely expressed in the brain mainly at the inhibitory interneurons. Electrophysiological studies on a recombinant *α*_1_ *β*_2_ *γ*_2_ GABA_A_ receptor showed no effect of *N*-methyllaurotetanine at concentrations lower than 30 *μ*M. However, (*S*)-reticuline behaved as positive allosteric modulator at the *α*_3_, *α*_5_, and *α*_6_ isoforms of GABA_A_ receptors. The depressant properties of aerial parts of *E. californica* are assigned to chloride-current modulation by (*S*)-reticuline at the *α*_3_ *β*_2_ *γ*_2_ and *α*_5_ *β*_2_ *γ*_2_ GABA_A_ receptors. Interestingly, *α*_1_, *α*_3_, and *α*_5_ were not significantly affected by (*R*)-reticuline, 1,2-tetrahydroreticuline, codeine, and morphine—suspected (*S*)-reticuline metabolites in the rodent brain.

## 1. Introduction

**Figure 1:**
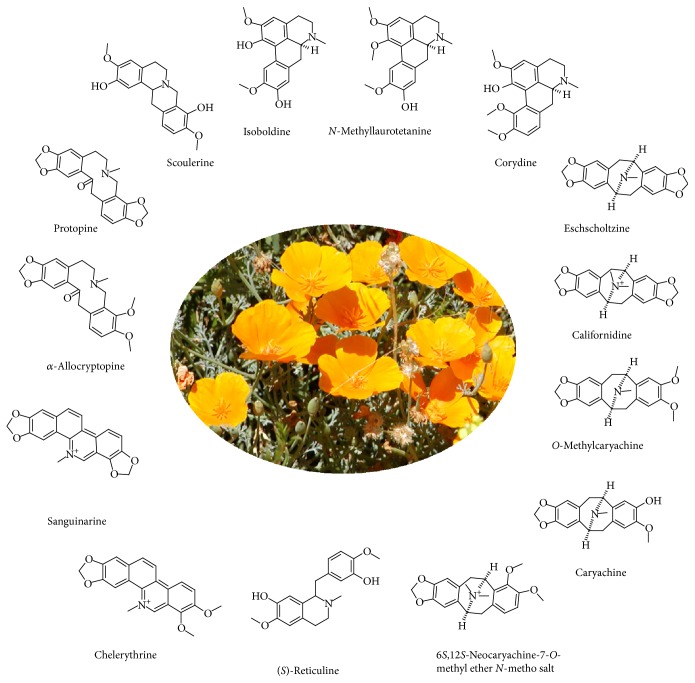
Alkaloids identified in the aerial parts of* Eschscholzia californica*.

The California poppy is known in folk medicine for its sedative, anxiolytic, and antinociceptive effects [1–4]. These effects have been traditionally assigned to protopine and allocryptopine (see Figure 1). Both alkaloids act as weak stimulators of the binding of GABA_A_ receptor agonists in the rat brain [5, 6], as anti-inflammatory agents [7] and as acetylcholinesterase inhibitors [7, 8]. Another aporphine alkaloid isolated from this plant, namely,* N*-methyllaurotetanine (NMT), was reported to act as antagonist at the serotonin 5HT_1A_R receptor (EC_50_ = 155 nM, *K*_*i*_ = 85 nM) [9]. Protopine and allocryptopine were also found to block human serotonin and noradrenaline transporters (hSERT and NERT) and possess antidepressant-like effects on animal models [10]. However, it is not clear whether typical pharmacy preparations (i.e., 300 mg of dry plant material per capsule) contain sufficient quantities of these alkaloids required to induce desired biological effects. Even though the presence of NMT in this plant has been clearly established, its content in the aerial parts of this perennial herb is currently unknown. Furthermore, it is not known whether or not this molecule interacts with the GABA_A_ receptor.

In the present work, we have studied total alkaloid extraction from aerial parts of* E. californica* by two methods. First, the standard chloroform/SDS method [11] relying on the ion pairing of alkaloids with an anionic detergent (Method A) was used. Second, the dry plant was macerated for extended period of time in methanol solutions, solution pH was adjusted, and alkaloids were extracted into diethylether [12] (Method B). Alkaloids contained in different fractions were analysed using the diode-array spectrophotometer coupled to HPLC and with the electrospray-tandem MS/MS spectrometry (ESI-MS/MS). The main goal of the present study was to identify the active principle in* E. californica* responsible for reported sedative and anxiolytic effects [1–4]. Finally, we would like to conclude with whether this medicinal herb has a potential of replacing some commonly used synthetic sleeping drugs and antidepressants.

## 2. Material and Methods

### 2.1. Chemicals

Protopine hydrochloride (≥98%) and *α*-allocryptopine (≥98%) were obtained from Sigma-Aldrich (Switzerland). (*S*)-(+)-Reticuline hydrochloride (95%, >90% ee), (*R*)-(−)-reticuline (95%), and 1,2-tetrahydroreticuline iodide (95%) used in electrophysiological experiments at the GABA_A_ receptors were obtained from Toronto Research Chemicals (Canada). Morphine sulphate salt pentahydrate (99.8%) and codeine certified reference material (1 mg/mL in methanol) were obtained from Sigma. Caryachine (98%) and O-methylcaryachine (98%) samples were kindly provided by Stefan Gafner (Tom's of Maine, USA).

### 2.2. Alkaloid Extraction Analysis

The alkaloid extraction procedure (Method A) employed in the present study was slightly modified as compared to the original protocol for the alkaloid extraction from* Chelidonium majus* [11].

*Method A*. Five grams of the dry plant material (*Eschscholzia californica*, Arcopharma, AMM 57426001, Switzerland) in a form of green powder was extracted into 100 mL of methanol (15 minutes at 60°C). Organic solvent (95 mL) was then distilled out at the rotary evaporator at the reduced pressure. Green-coloured solution was diluted with 50 mL of distilled water and solution pH was adjusted to 1.0 (drop-wise addition of 10% hydrochloric acid). Subsequently, 100 mg of 0.2 wt% sodium dodecyl sulfate (aq.) was added and alkaloids were extracted with chloroform (3 × 50 mL). The organic phase was dried using anhydrous Na_2_SO_4_ and the chloroform evaporated using the rotary evaporator at 40°C. Finally, the crude extract was dissolved in 10 mL MeOH and filtered using 5.0 *μ*m followed by 0.45 *μ*m PTFE microfilter. The plant extract was then subjected to a thin-layer chromatography (TLC) in methylene chloride/methanol (18 : 2) containing 0.1% trifluoroacetic acid as mobile phase. TLC spots were cut out using the razor blade, extracted into 5 mL methanol, and subjected to ESI-MS/MS analysis. Electrospray ionization high-resolution mass spectra were acquired with a FT/ICR mass spectrometer Bruker 4.7T BioApex II (Germany). Alkaloids were also separated and identified using the diode-array HPLC and commercial alkaloid standards. Figure 1 illustrates a typical alkaloid distribution in* E. californica* found in the present study using the HPLC and ESI-MS/MS (see Supplementary material available online at http://dx.doi.org/10.1155/2015/617620). 

**Figure 2:**
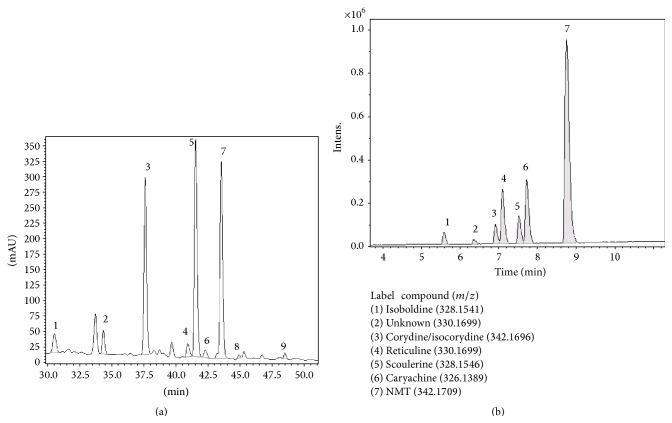
(a) LC UV chromatogram of* Eschscholzia californica *extract before fractionation. Peak identification: (1) reticuline; (2) caryachine; (3)* N*-methyllaurotetanine; (4) protopine; (5) californidine; (6) allocryptopine; (7) escholtzine; (8) sanguinarine; and (9) chelerythrine. (b) LC-MS trace of “NMT sample” containing 82%* N*-methyllaurotetanine, 10% reticuline, and 8% caryachine with traces of other alkaloids.

*Method B*.* E. californica* was obtained from Dixa AG (St Gallen, Switzerland) and a voucher sample was deposited at Interdelta (Givisiez, Switzerland) under the number 132914. The finely ground dry aerial part of* E. californica* (1.872 kg) was macerated in methanol for 10 weeks. Methanol was evaporated. The weight of dry extract was 412.5 g (22% of dry material). HPLC chromatogram of the extract before fractionation is shown in Figure 2(a). The dry plant extract was dissolved in 1% aq. H_2_SO_4_, aqueous phase was adjusted to pH *≅* 9 and alkaloid fraction was extracted into diethylether. Fraction A (8.06 g) was obtained once diethylether was distilled off. In order to isolate NMT, the plant sample was fractionated into two fractions: A1 (nonphenolic alkaloids) and A2 (phenolic alkaloids). Fraction A was dissolved in 1% H_2_SO_4_, pH was adjusted to 13, and nonphenolic alkaloids were extracted into diethylether (fraction A1). The remaining aqueous phase was acidified to pH 8 with H_2_SO_4_ and extracted second time into diethylether (fraction A2, phenolic). After evaporation of diethylether the mass of fraction A1 contained 4.19 g and the mass of the fraction A2 contained 2.02 g. Fraction A2 was found to contain 82% NMT, 10% reticuline, and 8% caryachine (Figure 2(b)). The method was described in our previous study [12]. Briefly, the mobile phase was prepared from a stock solution of 0.01 M sodium 1-heptanesulfonate and 0.1 M triethylamine in H_2_O, adjusted to pH 2.5 with phosphoric acid. Mobile phase A consisted of 25 : 75 (v/v) acetonitrile (ACN) :  stock solution. Mobile phase B was 60 : 40 (v/v) ACN : stock solution. Phosphoric acid and sodium 1-heptanesulfonate were obtained from Sigma-Aldrich (Prague, Czech Republic). ACN (HPLC grade) was purchased from Merck (Darmstadt, Germany). The following elution profile was employed: 0–4 min isocratically 100% A; 4–15 min 0–20% B; 15–38 min 45% B; 38–50 min 80% B; and 50–60 min isocratically 100% A. The flow rate was set to 0.5 mL/min, the injection volume was 100 *μ*L, and detection was performed using DAD (diode-array detector) at 280 nm. The identification of separated alkaloids was based on comparison of their retention times with those of authentic standards. Quantitative analysis was performed using external standards. The HPLC apparatus consisted of a LC-20 AD high-pressure gradient pump LC-20 AD, SPD M20A diode-array detector (Shimadzu, Japan) and a syringe-loading sample injector (ECOM, Czech Republic) with a 20-*μ*L external sample loop. Alkaloids were separated on a C12 column (Synergi RP-Max, 4 *μ*m, 150 × 4.60 mm ID, Phenomenex, CA).

The LC-MS method used to identify the “NMT fraction” alkaloids (Figure 2(b)) was developed using Dionex Ultimate 3000RS (Thermo Scientific, CA) module. Compound separation was achieved with 3.0 × 150 mm, 5 *μ*m Synergi RP-Max C18 (Phenomenex) column at 23°C and a flow rate of 0.5 mL/min. The binary mobile phase system consisted of 0.1% formic acid and LC-MS grade ACN (both Sigma). After 20-*μ*L injection, ACN was linearly increased from 20% to 40% over 10 min and then to 80% over the next 10 min. ACN was held at 80% for 10 min, followed by equilibration at the initial conditions for 3.0 min. A complete HPLC run was 33 min. The HPLC system was connected to a MicrOTOF-QII (Bruker, Germany) mass spectrometer, operated in positive electrospray ionisation mode. The ionisation conditions were set by the software as follows: the capillary voltage 4.5 kV, end plate offset −0.5 kV, source temperature 220°C, desolvation gas (nitrogen) flow 10 L/min, nebuliser (nitrogen) pressure 3 bar, and collision cell voltage 35 eV. The base-peak chromatogram (BPC) was acquired in MS mode by monitoring the range of 50 to 3000* m/z* with a spectra sample time of 1 s. The identification of target compounds relied on isotope pattern matching with a combination of MS/MS and retention behaviour. High-resolution MS and MS/MS spectra were first investigated to obtain the elemental formula of each compound. A compound was unambiguously identified if the fragmentation patterns of the unknown and the target alkaloid were identical.

### 2.3. Electrophysiological Experiments

Capped cRNAs were synthesized from the linearized plasmids. A poly-A tail of about 400 residues was added to each transcript using yeast poly-A polymerase. The concentration of the cRNA was quantified on a formaldehyde gel using Radiant red stain for visualization of the RNA. Known concentrations of RNA ladder were loaded as standard on the same gel. cRNAs were precipitated in ethanol/isoamylalcohol 19 : 1 and the dried pellet was dissolved in water and stored at −80°C.* Xenopus* oocytes (Centre de Ressources Biologiques Xénopes, UMS 3387, Université de Rennes, France. Animal research permit by the Kantonstierarzt, Kantonaler Veterinärdienst Bern (BE98/12)) were prepared, injected, and defolliculated as described previously [13]. Briefly,* Xenopus laevis* oocytes were injected with 50 nL of the cRNA solution containing *α*_1_, *α*_2_, *α*_3_, *α*_5_, or *α*_6_ in combination with *β*_1_, *β*_2_, or *β*_3_ and *γ*_2_ subunits at a concentration of 10 nM : 10 nM : 50 nM and then incubated in modified Barth's solution at 18°C for at least 24 h before the measurements. For *α*_1_ *β*_2_ *δ* 10 nM : 10 nM : 50 nM was used. Currents were measured using a home-built two-electrode voltage clamp amplifier in combination with XY-recorder or digitized using a PowerLab 2/20 (AD Instruments) using the computer program Chart. Tests with a model oocyte were performed to ensure linearity in the larger current range. The response was linear up to 15 *μ*A. Electrophysiological experiments were performed by using the two-electrode voltage clamp method at a holding potential of −80 mV. The perfusion medium contained 90 mM NaCl, 1 mM KCl, 1 mM MgCl_2_, 1 mM CaCl_2_, and 5 mM Na-HEPES (pH 7.4) and was applied by a gravity flow of 6 mL/min. Allosteric modulation was measured at a GABA concentration eliciting 0.5–1.5% of the maximal GABA current amplitude in the corresponding receptor. GABA was applied for 20 s alone or in combination with allosteric compound. The perfusion system was cleaned between drug applications by washing with DMSO to avoid contamination. Drugs were dissolved at 10 mM in DMSO and stored at −20°C. The final concentration of DMSO in the experiment was <0.5%. This concentration of DMSO did not affect currents elicited by GABA. The silicon-coated glass tubes were used for dilutions. These diluted solutions were always prepared fresh immediately before the experiment.

## 3. Results

### 3.1. Alkaloid Identification and Quantification

**Figure 3:**
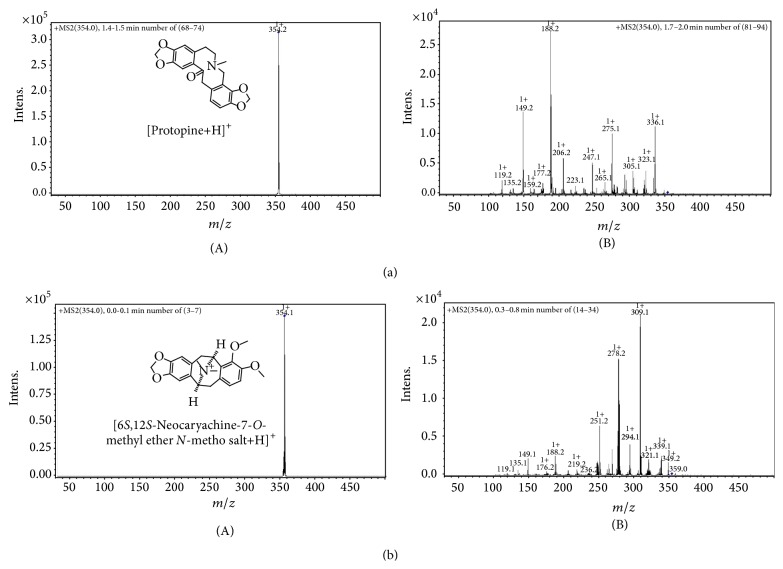
(a) (A) Electrospray ESI trace and (B) tandem MS/MS fragmentation pattern for selected ion* m/z* 354 corresponding to alkaloid protopine detected in a pharmacy capsule (300 mg) of* E. californica* (AMM 57426001) following alkaloid extraction and separation (Method A). (b) Electrospray ESI trace (A) and tandem MS/MS profile (B) for 6*S,*12*S*-neocaryachine-7-*O*-methyl ether* N*-metho salt detected in the same AMM 57426001 sample. Note that* m/z* 354 for this pavine alkaloid is practically identical to that of protopine; however, it gives quite different fragmentation pattern.

Figure 1 shows the chemical structure for all fourteen* E. californica* alkaloids identified in the present work. Nine of these were quantified using HPLC and appropriate alkaloid standards (Table 1), while the remaining compounds were positively identified by LC-MS and ESI-MS/MS (see Figures 2 and 3 and Figures S1–S8) and compared to previously reported MS data [9]. The advantage of this approach stems from the fact that each of studied alkaloids gives only one parent ion (+1) which can be further fragmented by tandem MS/MS so that each alkaloid had its own MS fingerprint signature and can be easily identified even though it is present in complex alkaloid mixtures (see Supplementary Material). It was suspected [12] that protopine and allocryptopine levels in the aerial parts of* E. californica* could be higher compared to relatively rapid alkaloid extraction (Method A) providing dry herb material would be macerated in methanol solutions for extended periods of time (Method B). It is evident from the data shown in Table 1 that even after 10 weeks of maceration the amount of protopine is rather low and about 43-fold higher than that of allocryptopine. On the other hand, and in contrast to rather low protopine yields, the NMT exceeds 11-fold amounts of protopine and its level is comparable to that of eschscholtzine. Unfortunately, the former compound was misinterpreted when analysing ESI-MS/MS data by Fabre et al. [14]. It is evident from the MS/MS fragmentation pattern shown in our Figure 3(b) that* m/z* 354.1 corresponds to this pavine alkaloid 6*S,*12*S*-neocaryachine-7-*O*-methyl ether* N*-metho salt rather than protopine—as claimed in Figure 5 by Fabre and coworkers [14]. Note that the molecular mass of both compounds is practically identical. This is rather unfortunate since their work, considered as reference in the area of alkaloid detection in* Papaveraceae *plants, actually missed to detect protopine—suggested as one of active principles in this medicinal herb. In addition to NMT, we have identified relatively high amount of reticuline (>1 mg/g of dry matter) in the aerial parts of* E. californica* (Table 1). This is quite important observation considering possible biological activity of this alkaloid (see below). Detection of reticuline in this plant was probably missed in older studies due to the lack of its separation from NMT at the HPLC columns used.

### 3.2. Biological Effects of* E. californica* Alkaloids

Protopine and allocryptopine are expected to act on GABA_A_ receptors in the micromolar range [5]. It is clear that levels of both alkaloids in* E. californica* analysed in the present study (Table 1) are relatively low and, therefore, submicromolar protopine levels in a single pharmacy capsule (300 mg) should fail to modulate the opening of the GABA_A_ receptors* in vitro *and* in vivo*. Since our* E. californica* extracts were found to contain relatively large amounts of NMT, it was suspected that this molecule could bind to GABA_A_ receptors and be responsible for sedation. Interestingly, the preliminary radiotelemetry experiments in C5BL/6J mice fed with the NMT-enriched fraction (82% NMT, 10% reticuline, and 8% caryachine with other minor alkaloids (Figure 2(a)) showed short-term sedative effects in a dose of 10 mg/kg between 30 and 54 min after oral administration (unpublished work by V. Butterweck, J. Wedler, University of Florida, Gainesville). In order to understand whether such sedation is due to NMT binding to GABA_A_ receptors, the electrophysiological studies were conducted using highly purified NMT (99.8%).

**Figure 4:**
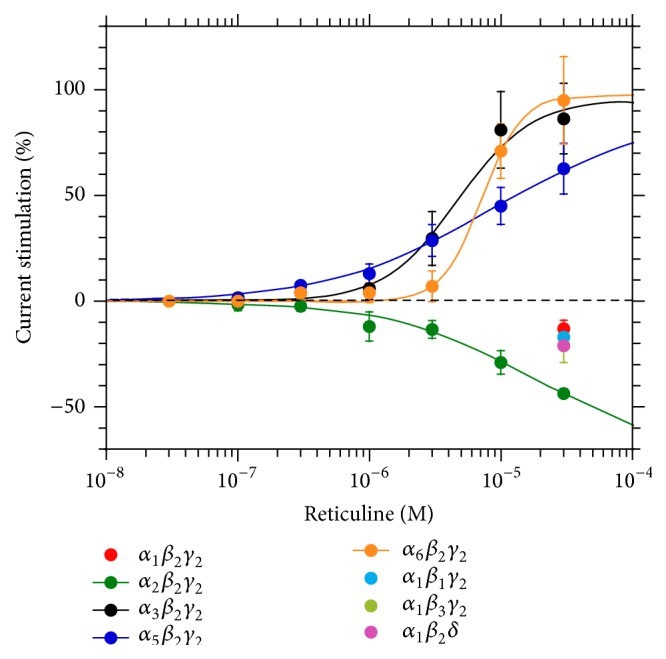
Modulation of recombinant GABA_A_ receptors of different subunit composition by (*S*)-reticuline. Receptors were expressed in* Xenopus* oocytes and concentration dependent modulation was determined using electrophysiological techniques. Each curve is the average of three to four determinations (data points ± standard deviation).

**Figure 5:**
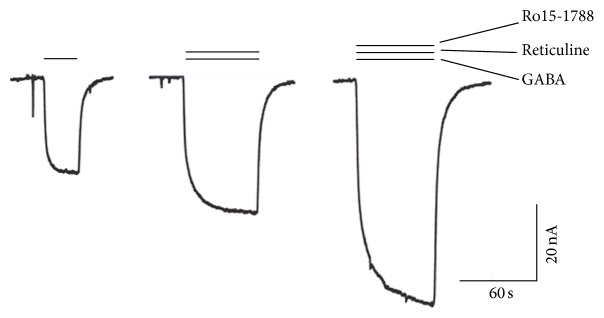
Effect of a benzodiazepine antagonist on the potentiation of *α*_5_ *β*_2_ *γ*_2_ GABA_A_ receptors by (*S*)-reticuline. Application of 0.5 *μ*M GABA was followed by application of 0.5 *μ*M GABA/10 *μ*M (*S*)-reticuline and subsequently by 0.5 *μ*M GABA/10 *μ*M (*S*)-reticuline/1 *μ*M Ro15-1788. Providing (*S*)-reticuline would act at the benzodiazepine site elicited currents would be expected to go back to the size elicited by GABA alone. Instead, a potentiation was observed. Similar observations were made in two additional experiments.

*E. californica* alkaloid binding on GABA_A_ receptors (*in vitro*) was not studied so far because of the presence of *γ*-aminobutyric acid in the plant extracts [9]. Therefore, we have studied effects of pure NMT on recombinant GABA_A_ receptors using electrophysiological technique. Typically, four milligrams of highly purified NMT was dissolved in a stock DMSO solution and appropriate dilutions were made for titrations at recombinant *α*_1_ *β*_2_ *γ*_2_ GABA_A_ receptors. Interestingly, NMT titrations have revealed no allosteric modulation up to 30 *μ*M. Stimulation by 1 *μ*M diazepam was not decreased by 10 *μ*M NMT, excluding an antagonistic effect at the benzodiazepine binding site. Similarly, protopine had no significant effects up to a concentration of 30 *μ*M. Additional experiments were performed with (*S*)-reticuline HCl salt (>90% isomer provided by Toronto Research Chemicals). Again, no significant effects of this molecule on recombinant *α*_1_ *β*_2_ *γ*_2_ receptors were noticed for concentrations below 10 *μ*M. Instead, about 28% inhibition of chloride currents was observed in the presence of 30 *μ*M (*S*)-reticuline (Figure 4). A similar behaviour was observed upon exchanging *β*_2_ subunit by *β*_1_ or *β*_3_ and in *α*_1_ *β*_2_ *δ* receptors (Figure 4). Excitingly, (*S*)-reticuline performed as a positive allosteric modulator at *α*_3_ *β*_2_ *γ*_2_, *α*_5_ *β*_2_ *γ*_2_, and *α*_6_ *β*_2_ *γ*_2_ receptors. Maximal stimulation was in each case about 100% of the maximal GABA effect and half-maximal stimulation (EC_50_) was observed at about 6 *μ*M. In contrast at *α*_2_ *β*_2_ *γ*_2_ receptors we observed allosteric inhibition (Figure 4). 10 *μ*M (*S*)-reticuline did not by itself elicit currents in *α*_3_ *β*_2_ *γ*_2_ receptors. As in the case of *α*_1_ *β*_2_ *γ*_2_ receptor, only a small inhibition was observed at high concentration at *α*_1_ *β*_1_ *γ*_2_, *α*_1_ *β*_3_ *γ*_2_, and *α*_1_ *β*_2_ *δ* receptors. While (*R*)-reticuline had no effect (see below), the modulatory effect by (*S*)-reticuline was stereospecific. As Figure 5 documents, 1 *μ*M Ro15-1788, an antagonist acting at the benzodiazepine binding site, did not inhibit the potentiation by 10 *μ*M (*S*)-reticuline in *α*_3_ *β*_2_ *γ*_2_ receptors but rather stimulated its action. This indicates that (*S*)-reticuline does not act at the benzodiazepine binding site. It may be speculated that (*S*)-reticuline contacts at least one amino acid residue of the *α* subunit that is similar in *α*_3_, *α*_5_, and *α*_6_ but different from the homologous residue in *α*_2_ and both must be different from the homologous residue in *α*_1_. Alignment of the sequences of all *α* subunits reveals that only few positions qualify as part of the binding site.

Suspected (*S*)-reticuline metabolites in the rodent organism were also tested for modulation. Interestingly, recombinant *α*_1_ *β*_2_ *γ*_2_, *α*_3_ *β*_2_ *γ*_2_, and *α*_5_ *β*_2_ *γ*_2_ GABA_A_ receptors were not significantly affected by 10 *μ*M of either (*R*)-reticuline, 1,2-tetrahydroreticuline, codeine, or morphine (not shown).

## 4. Discussion

During the last three decades, several analytical studies have addressed the issues of alkaloid detection and quantification in green parts of* E. californica* [14–16]. Unfortunately, significant differences in the alkaloid content have been noticed. For example, protopine levels were found to vary by two orders of magnitude going from traces [9, 14, 16] to comparable levels to those of californidine and eschscholtzine [15]—two most abundant alkaloids in this plant. In order to make sure that the majority of alkaloids get extracted from the dried plant tissues, we have conducted their maceration in methanol up to 10 weeks, which was followed by alkaloid quantification by diode-array HPLC. We were especially interested in protopine and allocryptopine levels since both alkaloids were suggested to act at the GABA_A_ receptor [5, 6].

The electrophysiological experiments conducted in the present work at various GABA_A_ receptor isoforms suggest that protopine does not play significant role regarding the sedative effects of* E. californica* reported in previous studies. Typical recommendation for the* E. californica* dose is two 300 mg capsules of dry plant material before sleep (https://www.swissmedic.ch/zulassungen/00153/00189/00200/01041/index.html?lang=de). It is evident from HPLC analysis (Table 1) that protopine and *α*-allocryptopine levels in the aerial parts of this herb are too low to modulate significantly the chloride-ion flow across the GABA_A_ receptors. Both protopine and allocryptopine are known as inhibitors of the human serotonin and noradrenaline transporters [10]; however, 0.01–0.5 mg/g of these alkaloids is certainly too low to boost the serotonin or noradrenaline levels in depressed patients. Hanus and colleagues [4] have conducted double-blind placebo-controlled study to evaluate the efficacy and safety of* E. californica* in combination with* Crataegus oxyacantha *extracts for the treatment of mild-to-moderate anxiety disorders. However,* Crataegus* is known to contain several flavonoids such as quercetin and rutin, flavonol kaempferol, and other compounds known to act on the central nervous system [17]. Quercetin and kaempferol have been reported to bind at the GABA_A_ receptors [18], which was suggested to explain sedative and anxiolytic effects of these compounds [18–20]. Quercetin also agonizes 5HT_1A_ receptor* in vitro* and it has been suggested as a candidate to explain its antinociceptive properties [21]. Even though several flavone glycosides of quercetin, rutin, and isorhamnetin were previously detected in* E. californica* [22], we could detect only traces of kaempferol in aerial parts of this plant (HPLC data not shown). However, NMT appears as a relatively strong antagonist with respect to the 5HT_1A_ receptor [9] and, as the result, it is unlikely to promote antinociceptive effects of* E. californica*. Therefore, there must exist another mechanism of how this plant affects the central nervous system in mammals. Interestingly, we have shown in the present work that aerial parts of* E. californica* contain (*S*)-reticuline. It has been shown [23] that this compound may be transformed by neuroblastoma cells into morphine, which is known to bind to *μ*-opioid receptors. The latter observation could explain why pure (*S*)-reticuline isolated from* Ocotea duckei* acted as potent central nervous system depressant [24].

Telemetry experiments with 10% (*S*)-reticuline, present in the NMT fraction, showed sedative effects on mice. However, our electrophysiological experiments have clearly established that there is no positive modulation of chloride currents by (*S*)-reticuline at the recombinant *α*_1_ *β*_2_ *γ*_2_ GABA_A_ receptors, the main isoform in the brain thought to mediate sedative effects. Therefore, (*S*)-reticuline is likely to get metabolized in rodents to some other compound manifesting significantly higher modulation of GABA_A_ receptors than the parent molecule. It has been reported [25] that (*S*)-reticuline may be metabolized in rodents via 1,2-dehydroreticuline, (*R*)-reticuline, and other alkaloid intermediates into codeine and morphine. In this respect, Nikolaev and colleagues [26] monitored G_*i*_-protein activation in living HEK293a cells expressing human *μ*-opioid receptors (MOR) and tested binding of morphine and codeine. The binding constant at MOR was determined in the nanomolar range (*K*_*i*_ = 4 ± 1 nM), with EC_50_ = 6 ± 2 nM for G_*i*_ activation by morphine. Codeine appeared to have much lower antinociceptive potency with both the *K*_*i*_-constant and EC_50_ of 6 *μ*M. Both of these molecules are also formed in* Papaveraceae* family plants via successive biochemical transformations of (*S*)-reticuline—the morphine being the final alkaloid product [25].

Immunoreactivity staining of the adult mouse brain colocalized endogenous morphine and codeine with GABAergic interneurons and astrocytes [27]. Morphine concentrated in a small population of neurones could function as a neuromodulator able to affect indirectly the inhibitory activity of GABAergic neurons. In particular, parvalbumin- (PV-) positive terminals of basket cells (GABAergic interneurons) express *μ*-opioid receptors and M2 muscarinic receptors, targeting the somatic part of pyramidal neurons equipped with *α*_1_ subunit-containing GABA_A_ receptors [28, 29]. This is in contrast to cholecystokinin- (CCK-) positive interneurons—expressing mainly cannabinoid CB1 and estrogen and metabotropic GABA_B_ receptors. Since alkaloids coming from (*S*)-reticuline biotransformation are able to bind to *μ*-opioid receptors, this could in turn affect GABA release at the PV terminals of the inhibitory cells and be responsible for mild sedative effects observed in our telemetric experiments. Providing there is even distribution and little degradation of (*S*)-reticuline, the level of 3 *μ*M in the rodent brain may be estimated. This is also high enough to affect *α*_3_ *β*_2_ *γ*_2_ and *α*_5_ *β*_2_ *γ*_2_ GABA_A_ receptors. The rat brain level of (*S*)-reticuline has been estimated as 13 ng/g [30], corresponding to an average concentration of about 0.05 *μ*M. On the other hand, only a small fraction of the neurons contains morphine metabolites—that is, mainly GABAergic neurons [31]. Therefore such a reticuline metabolite-dependent modulation of inhibitory interneurons may be relevant to* in vivo* situation.

## 5. Conclusion

ESI-tandem MS/MS and HPLC allowed us to detect, identify, and quantify majority of alkaloids present in* E. californica*. Modulation of chloride currents by protopine, *α*-allocryptopine, NMT, and reticuline at different recombinant GABA_A_ receptor isoforms was studied* in vitro*. Our choice of studying NMT and reticuline comes from the fact that the “NMT fraction” (see Section 3.2) manifested mild sedative effects on mice. On the other hand, much more abundant californidine (a quaternary pavine alkaloid) is unable to penetrate the brain-blood barrier [32] and, therefore, was not included in our electrophysiological study. The main goal of the present work was to identify the mechanism by which* E. californica* alkaloids could induce sedative effects. The main finding of the present work is that sedation caused by this medicinal herb does not depend on direct binding of alkaloids to GABA_A_ receptors and chloride current modulation. Even though (*S*)-reticuline was found to act on *α*_3_ *β*_2_ *γ*_2_ and *α*_5_ *β*_2_ *γ*_2_ GABA_A_ receptors, eventually, it may get transformed in the mammal body into more potent alkaloids which are then able to bind to GPCR receptors in the nanomolar range. We believe that mild sedative and antinociceptive properties of dry aerial parts of* E. californica* may be assigned to binding of morphine (and/or other alkaloids) at the *μ*-opioid receptors. Morphine may be generated in the rodent organism via successive biotransformation of (*S*)-reticuline present in aerial parts of* E. californica*. The presence of this alkaloid in aerial parts of California poppy was probably missed in previous studies since tis molecule probably coeluted with NMT under typical HPLC conditions [14].

In conclusion, in order to achieve important medicinal effects (regarding relatively low alkaloid levels determined in aerial parts of this plant), one would need either to increase the dried plant dosage above 1 g before sleep, or, eventually, to combine* E. californica* with other medicinal herbs containing other sleep-promoting alkaloids.

## Supplementary Material

Figure S1. ESI trace (a) and tandem MS/MS fragmentation pattern (b-c) for (S)-Reticuline detected in the “NMT fraction” isolated from *E. californica* (see the main text).
Figure S2. ESI trace (a) and tandem MS/MS fragmentation pattern (b) for Californidine isolated from *E. californica*, Arcopharma, no. AMM 57426001 (Method A).
Figure S3. ESI trace (a) and tandem MS/MS fragmentation pattern (b) for Eschscholtzine isolated from *E. californica*, Arcopharma, no. AMM 57426001 (Method A).
Figure S4. ESI trace (a) and tandem MS/MS fragmentation pattern (b) for *O*-methylcaryachine isolated from *E. californica*, Arcopharma, no. AMM 57426001 (Method A).
Figure S5. ESI trace (a) and tandem MS/MS fragmentation pattern (b) for α-allocryptopine isolated from *E. californica*, Arcopharma, no. AMM 57426001 (Method A).
Figure S6. ESI trace (a) and tandem MS/MS fragmentation pattern (b) for *N*-methyllaurotetanine isolated from *E. californica*, Arcopharma, no. AMM 57426001 (Method A).
Figure S7. ESI trace (a) and MS/MS fragmentation pattern (b) for the commercial Protopine sample obtained from Sigma (product number: P8489).
Figure S8. ESI trace (a) and MS/MS fragmentation pattern (b) for the commercial α-allocryptopine obtained from Aldrich (product number: S450987).

## Figures and Tables

**(a):** 

	Alkaloid (mg/g)						
	NMT	PRO	CAL	ALL	ESCH	SA	CHE
CH_3_OH	0.702 ± 0.159	0.137 ± 0.057	2.413 ± 0.331	0.035 ± 0.016	0.902 ± 0.074	0.041 ± 0.002	0.019 ± 0.003
CHCl_3_-SDS	0.557 ± 0.138	0.136 ± 0.113	1.951 ± 0.415	0.037 ± 0.010	0.545 ± 0.086	0.040 ± 0.015	0.022 ± 0.100

**(b):** 

Alkaloid	(mg/g)
Protopine (PRO)	0.514 ± 0.038
Californidine (CAL)	12.5 ± 1.8
Allocryptopine (ALL)	0.0120 ± 0.0023
Eschscholtzine (ESCH)	8.700 ± 0.51
Sanguinarine (SA)	0.0191 ± 0.0050
Chelerythrine (CHE)	0.068 ± 0.011
Reticuline (RE)	1.095 ± 0.16
N-Methyllaurotetanine (NMT)	5.68 ± 0.72
Caryachine (CAR)	0.410 ± 0.065
