# Supplementary material for: Modulatory Effects of Eschscholzia californica Alkaloids on Recombinant GABAA Receptors
Source: Biochem Res Int. 2015 Oct 5;2015:617620. doi: 10.1155/2015/617620 (PMC4609799; doi:10.1155/2015/617620)
Supplement: Supplementary file 1 — Figure S1. ESI trace (a) and tandem MS/MS fragmentation pattern (b-c) for (S)-Reticuline detected in the “NMT fraction” isolated from E. californica (see the main text). Figure S2. ESI trace (a) and tandem MS/MS fragmentation pattern (b) for Californidine isolated from E. californica, Arcopharma, no. AMM 57426001 (Method A). Figure S3. ESI trace (a) and tandem MS/MS fragmentation pattern (b) for Eschscholtzine isolated from E. californica, Arcopharma, no. AMM 57426001 (Method A). Figure S4. ESI trace (a) and tandem MS/MS fragmentation pattern (b) for O-methylcaryachine isolated from E. californica, Arcopharma, no. AMM 57426001 (Method A). Figure S5. ESI trace (a) and tandem MS/MS fragmentation pattern (b) for α-allocryptopine isolated from E. californica, Arcopharma, no. AMM 57426001 (Method A). Figure S6. ESI trace (a) and tandem MS/MS fragmentation pattern (b) for N-methyllaurotetanine isolated from E. californica, Arcopharma, no. AMM 57426001 (Method A). Figure S7. ESI trace (a) and MS/MS fragmentation pattern (b) for the commercial Protopine sample obtained from Sigma (product number: P8489). Figure S8. ESI trace (a) and MS/MS fragmentation pattern (b) for the commercial α-allocryptopine obtained from Aldrich (product number: S450987). [file 617620.f1.pdf]

(in $CHCl_3$ + MeOH) Instrument: BRUKER - Ion Trap MS esquire HC

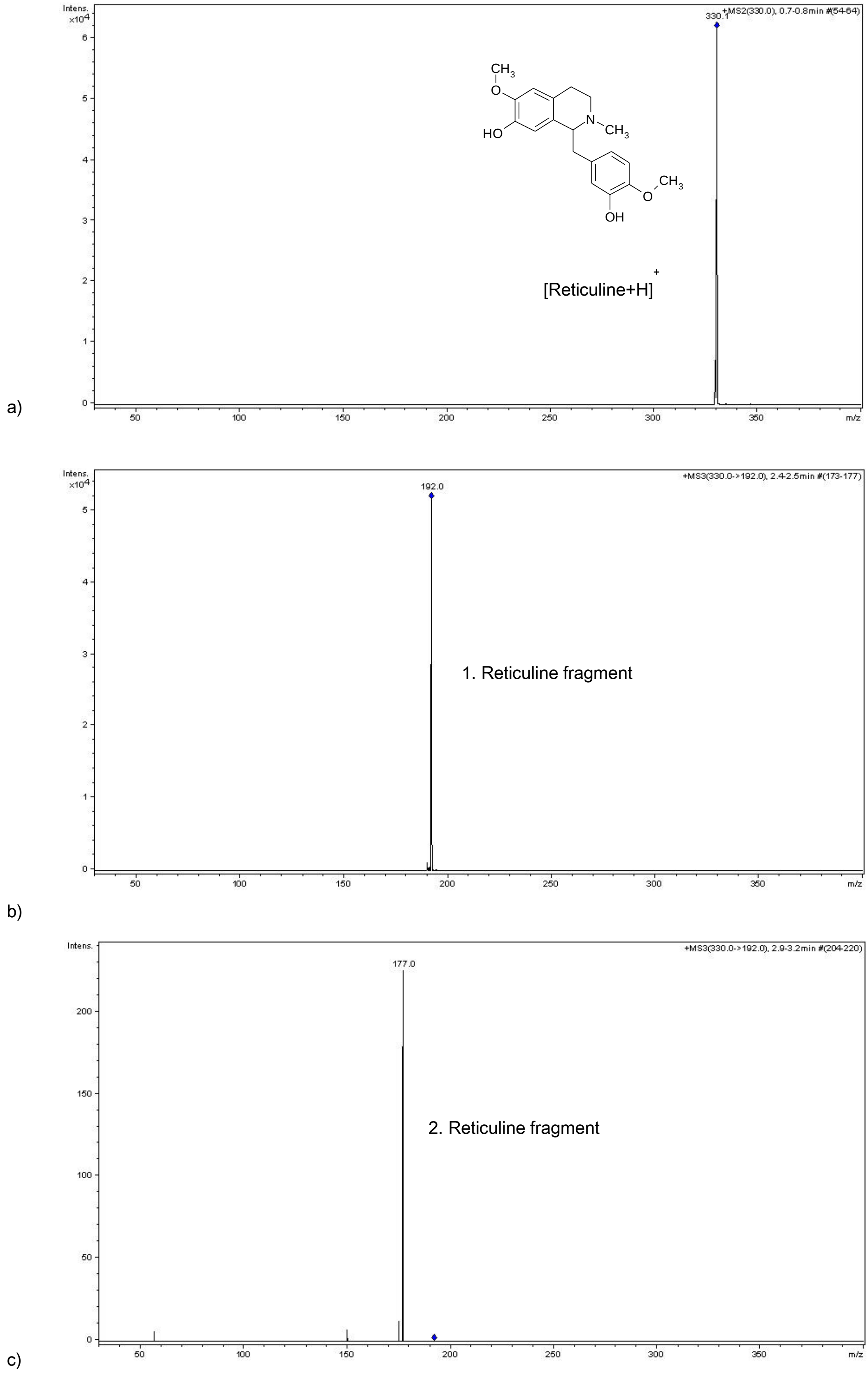

Figure S1. ESI trace (a) and tandem MS/MS fragmentation pattern (b-c) for (*S*)-Reticuline detected in the “NMT fraction” isolated from *E. californica* (see the main text).

Instrument: BRUKER - Ion Trap MS esquire HC

(in MeOH)

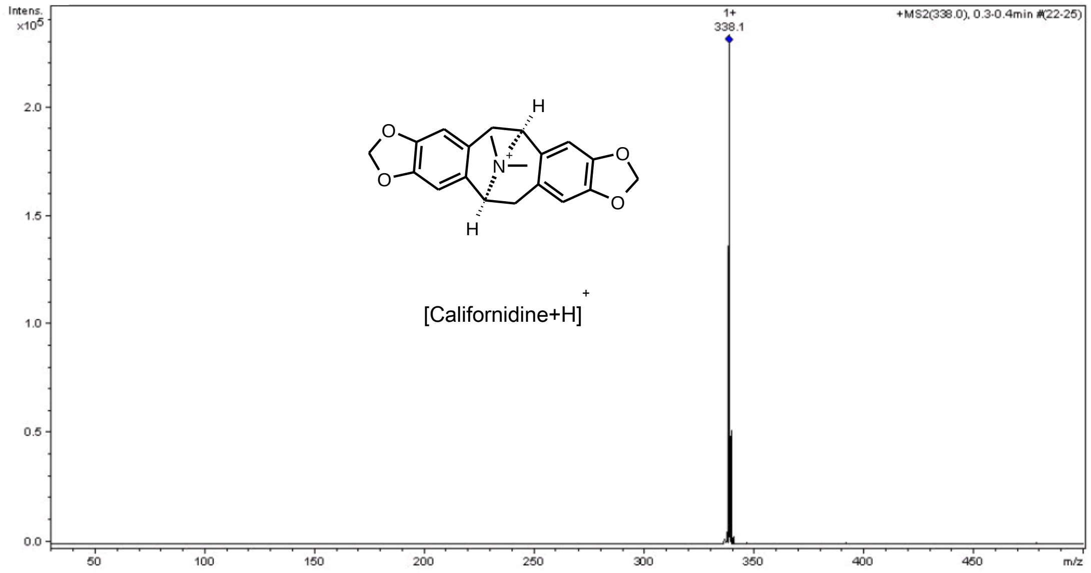

a)

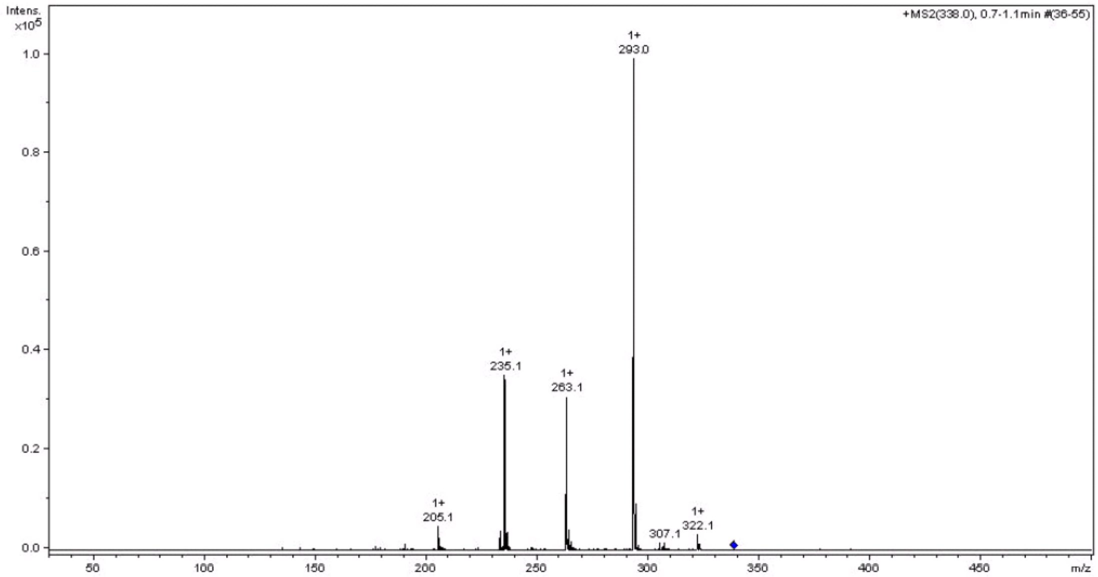

b)

Figure S2. ESI trace (a) and tandem MS/MS fragmentation pattern (b) for Californidine isolated from *E. californica*, Arcopharma, no. AMM 57426001 (Method A).

(in MeOH)

Instrument: BRUKER - Ion Trap MS esquire HC

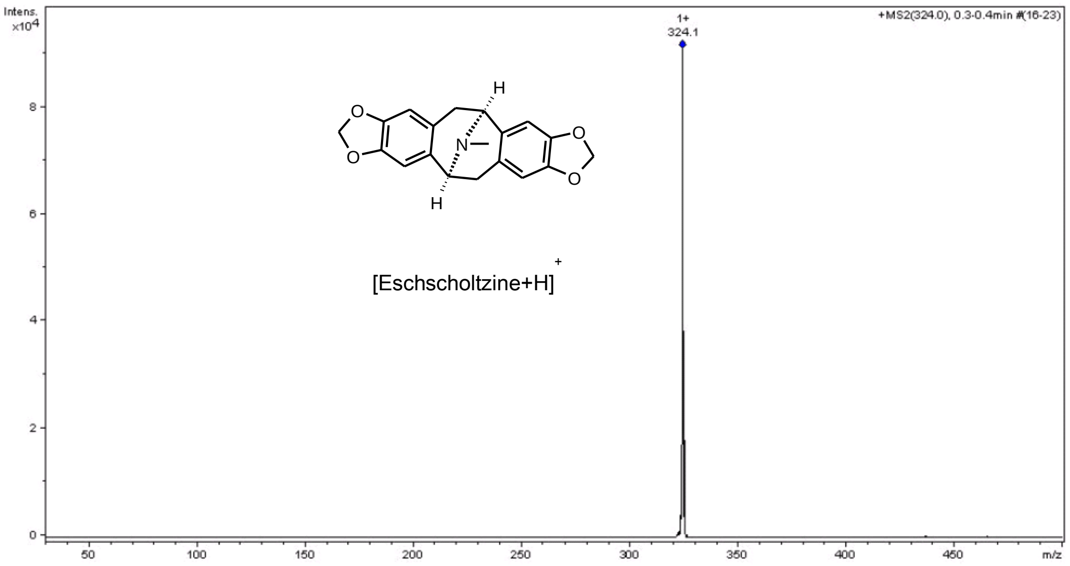

a)

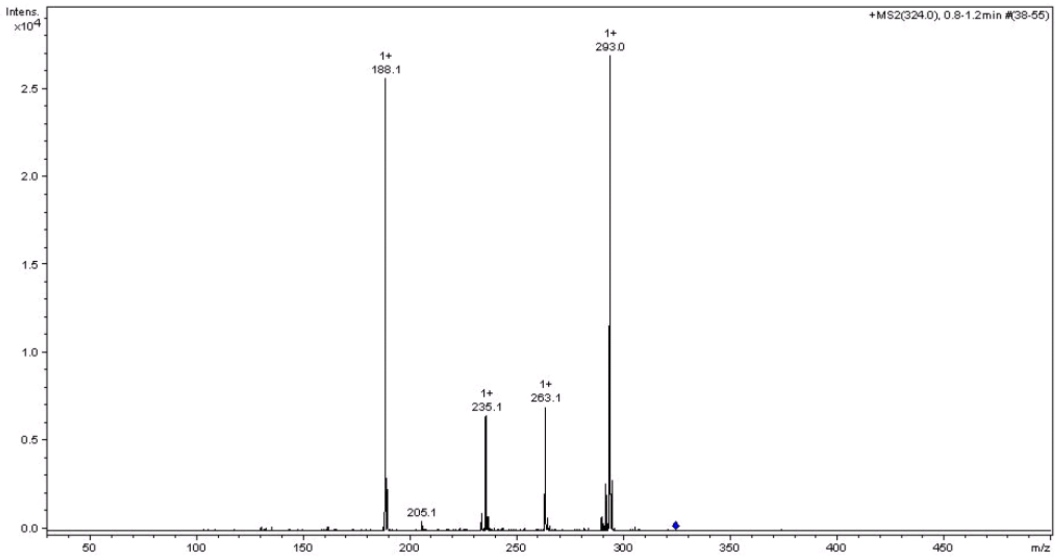

b)

Figure S3. ESI trace (a) and tandem MS/MS fragmentation pattern (b) for Eschscholtzine isolated from *E. californica*, Arcopharma, no. AMM 57426001 (Method A).

(in MeOH) Instrument: BRUKER - Ion Trap MS esquire HC

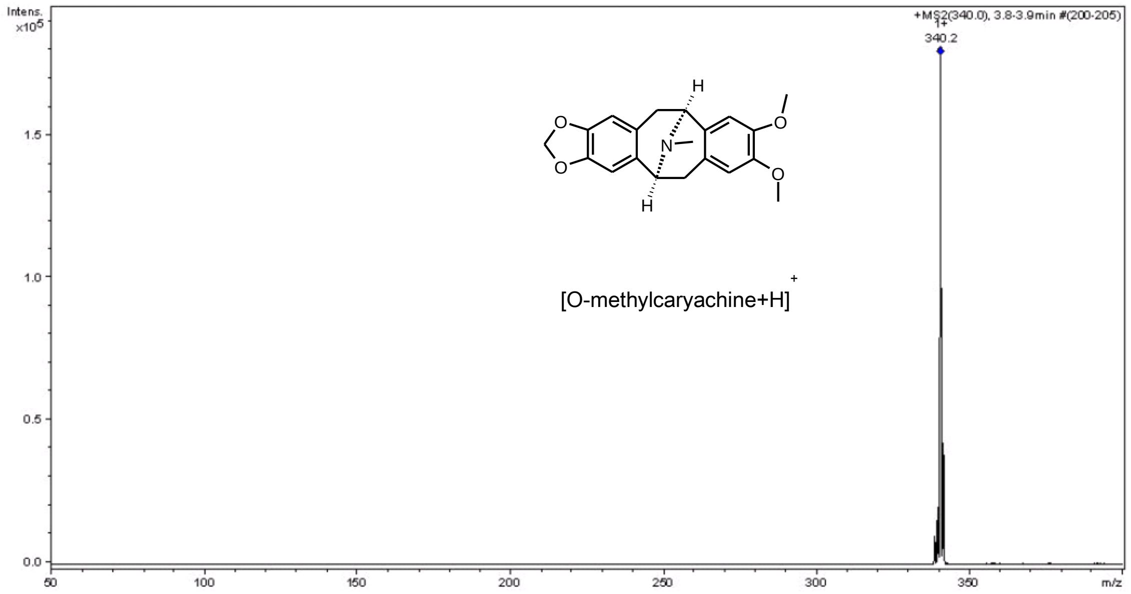

a)

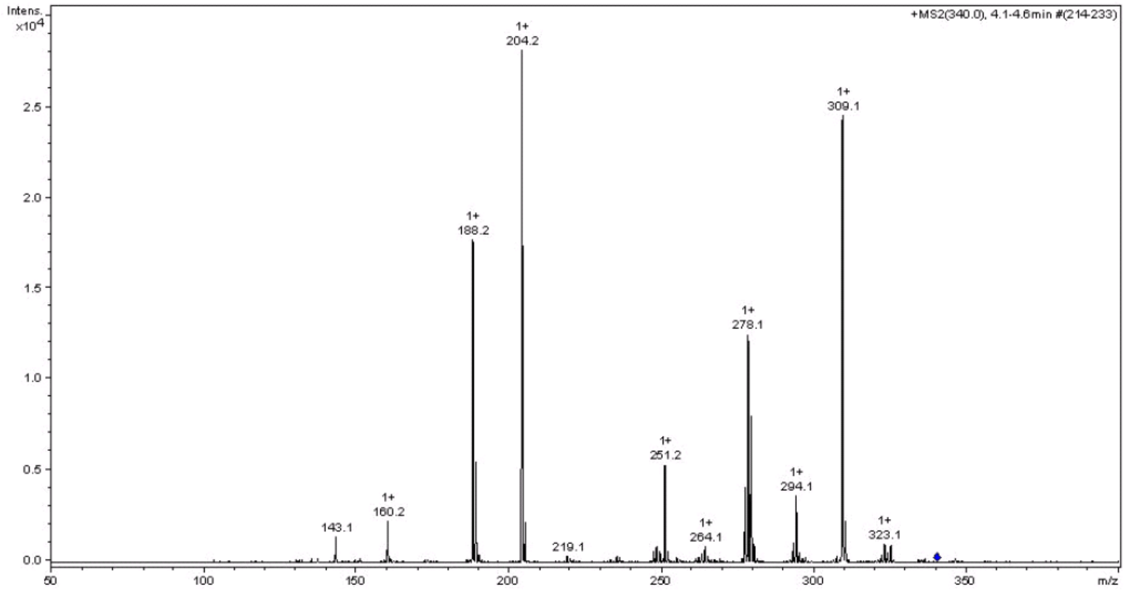

b)

Figure S4. ESI trace (a) and tandem MS/MS fragmentation pattern (b) for *O*-methylcaryachine isolated from *E. californica*, Arcopharma, no. AMM 57426001 (Method A).

(in MeOH) Instrument: BRUKER - Ion Trap MS esquire HC

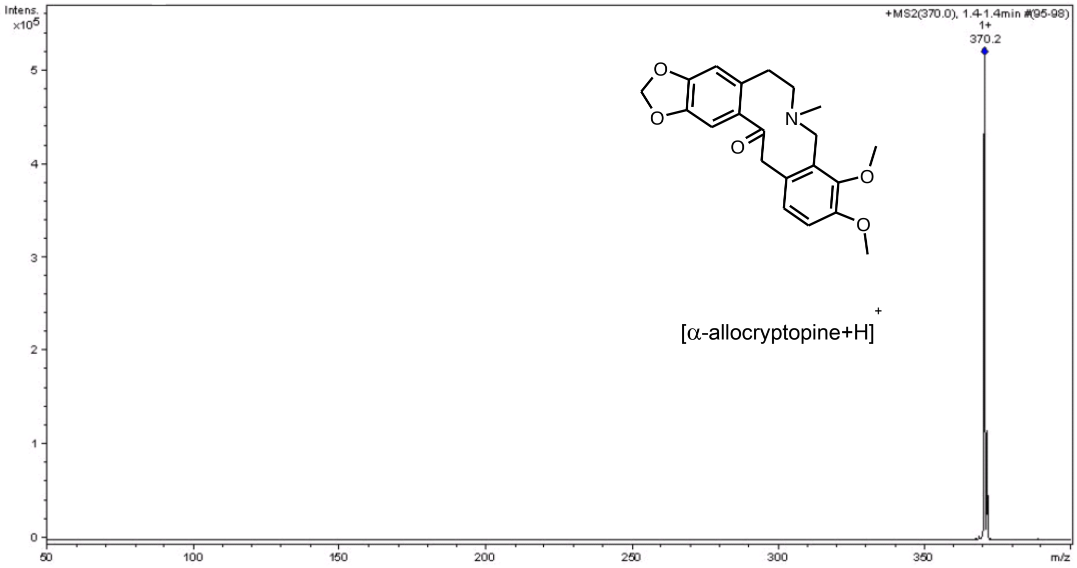

a)

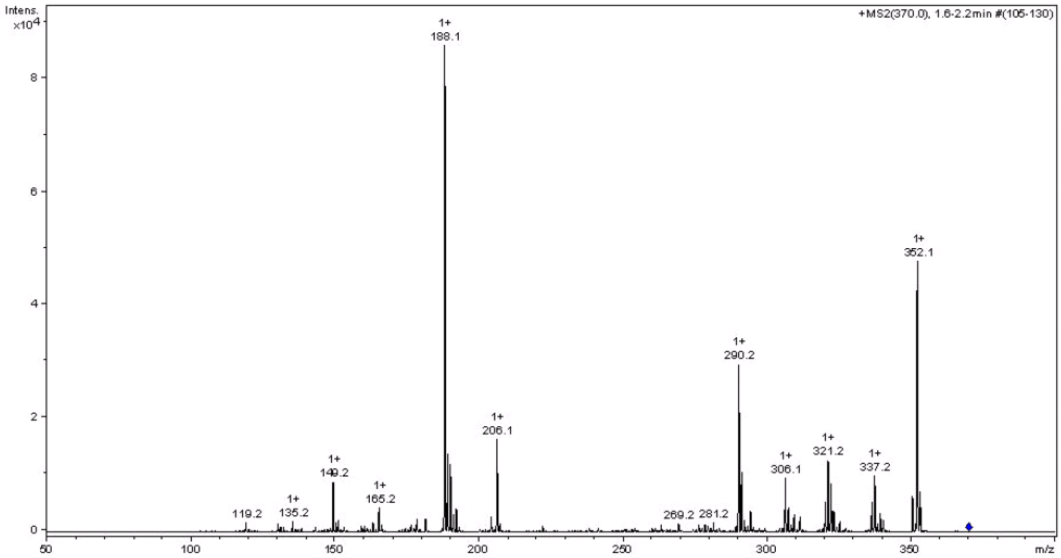

b)

Figure S5. ESI trace (a) and tandem MS/MS fragmentation pattern (b) for α-allocryptopine isolated from *E. californica*, Arcopharma, no. AMM 57426001 (Method A).

(in MeOH) Instrument: BRUKER - Ion Trap MS esquire HC

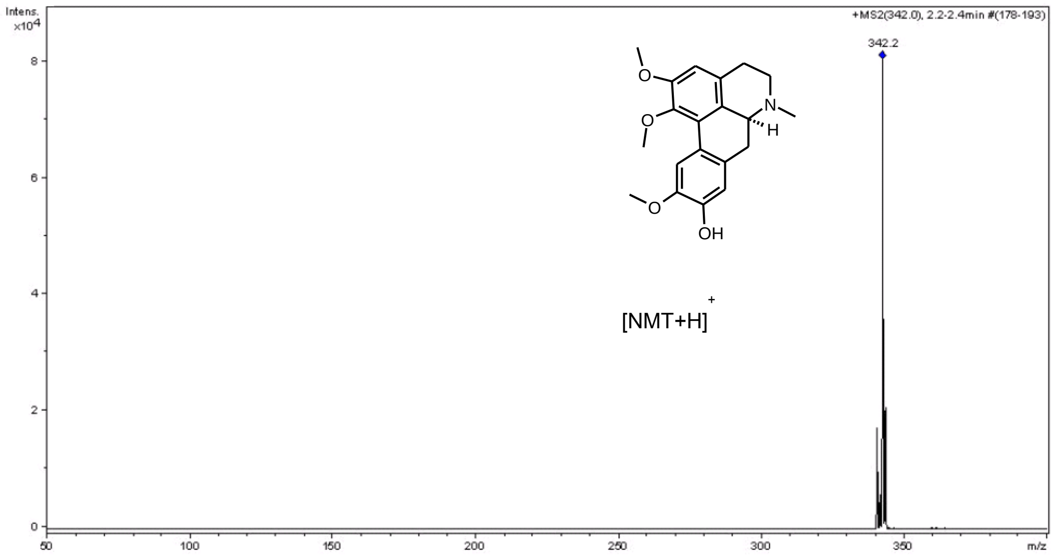

a)

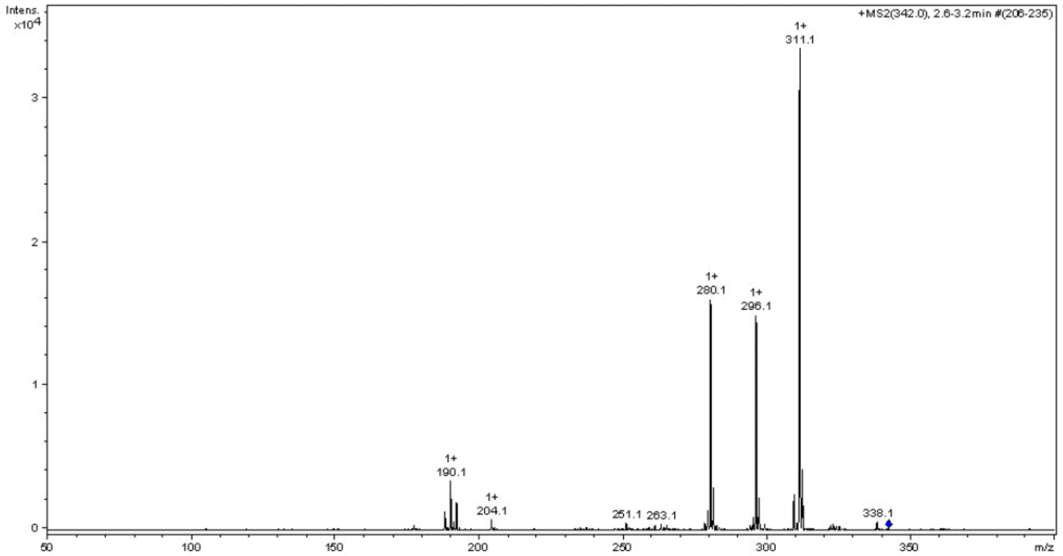

b)

Figure S6. ESI trace (a) and tandem MS/MS fragmentation pattern (b) for *N*-methyllaurotetanine isolated from *E. californica*, Arcopharma, no. AMM 57426001 (Method A).

(in MeOH) Instrument: BRUKER - Ion Trap MS esquire HC

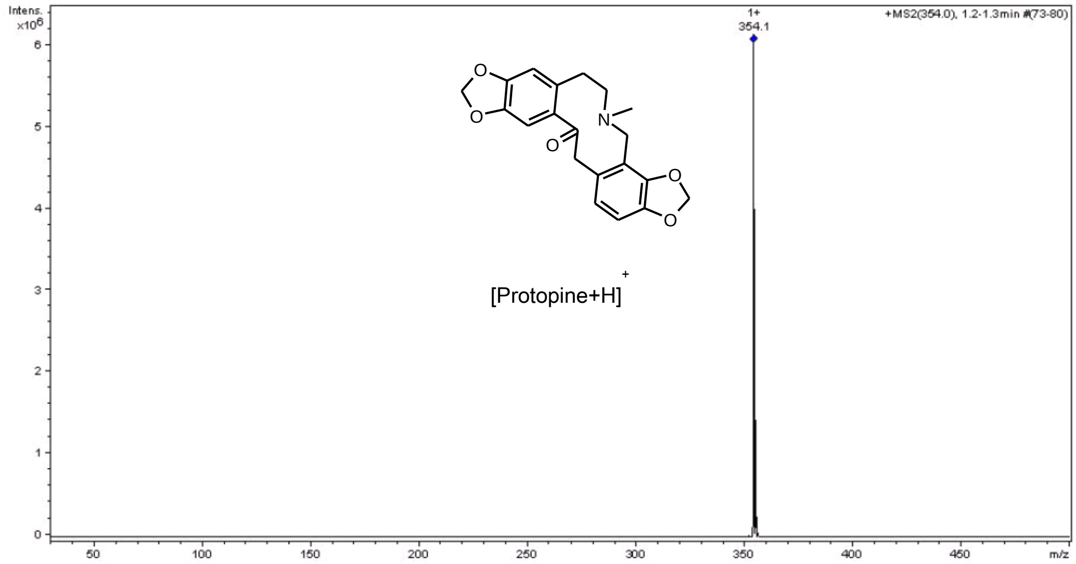

a)

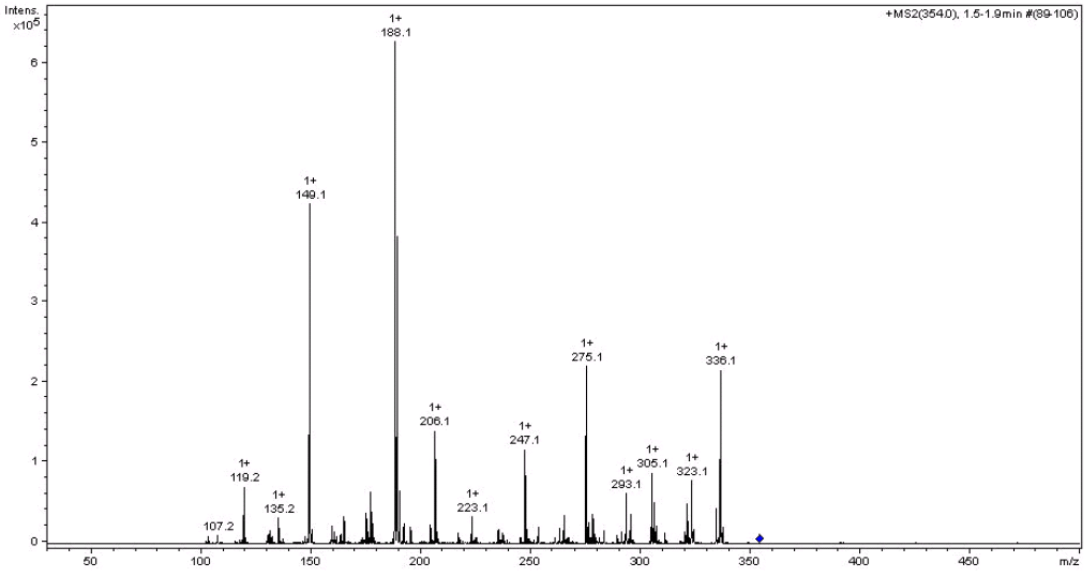

b)

Figure S7. ESI trace (a) and MS/MS fragmentation pattern (b) for the commercial Protopine sample obtained from Sigma (product number: P8489).

(in MeOH) Instrument: BRUKER - Ion Trap MS esquire HC

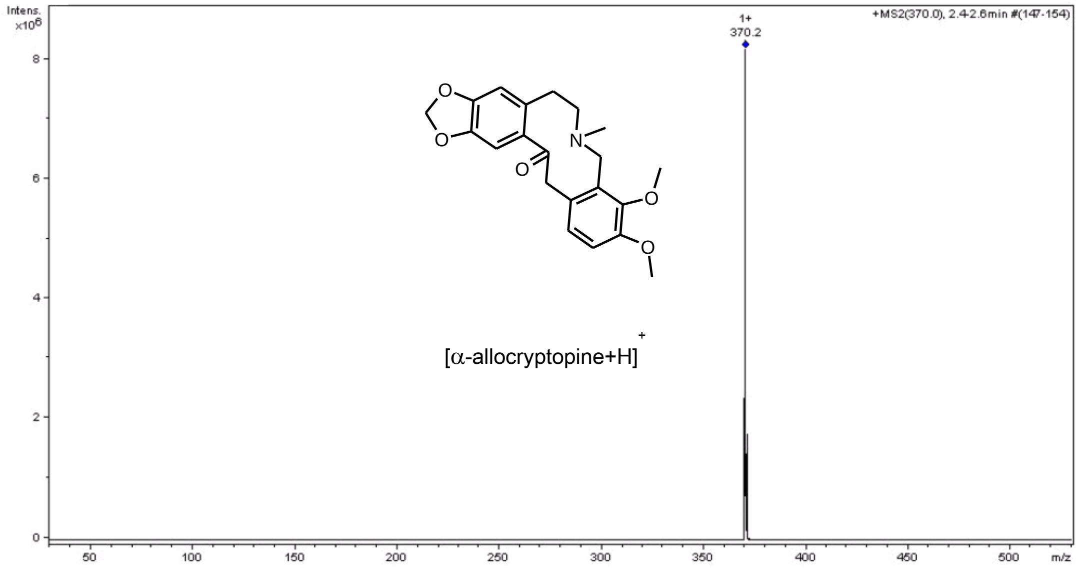

a)

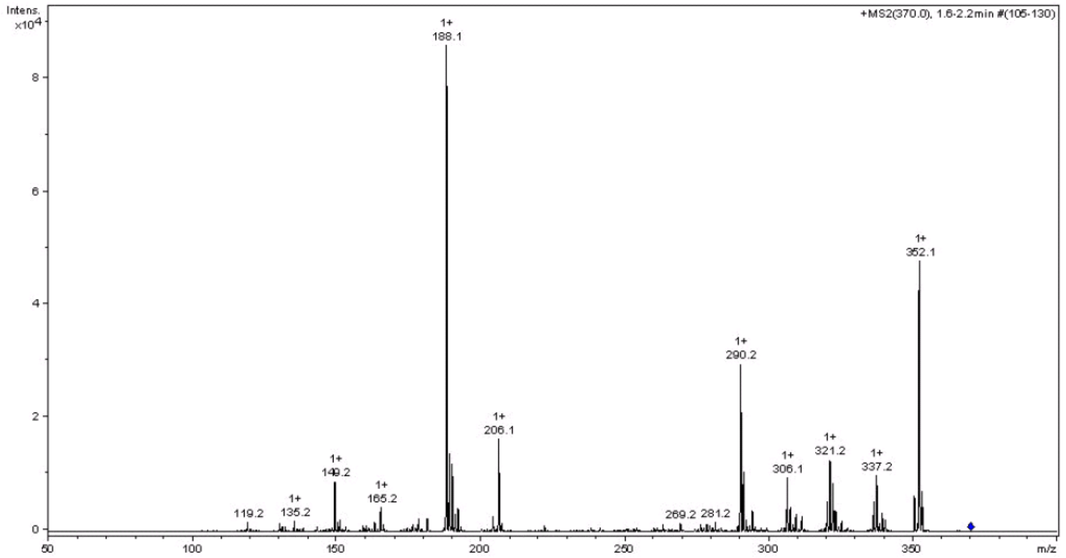

b)

Figure S8. ESI trace (a) and MS/MS fragmentation pattern (b) for the commercial α-allocryptopine obtained from Aldrich (product number: S450987).
